# Endoskopische Unterdrucktherapie: Vom Komplikationsmanagement zur präemptiven aktiven Refluxdrainage bei abdomino-thorakaler Ösophagusresektion – ein neues Sicherheitskonzept für die Ösophaguschirurgie

**DOI:** 10.1007/s00104-023-01970-2

**Published:** 2023-10-26

**Authors:** Gunnar Loske, Johannes Müller, Wolfgang Schulze, Burkhard Riefel, Matthias Reeh, Christian Theodor Müller

**Affiliations:** 1 Klinik für Allgemein‑, Viszeral‑, Thorax- und Gefäßchirurgie, Katholisches Marienkrankenhaus Hamburg, Alfredstr. 9, 22087 Hamburg, Deutschland; 2 Chirurgie, Wilhelmsburger Krankenhaus Groß-Sand, Groß-Sand 3, 21107 Hamburg, Deutschland

**Keywords:** Intraluminale endoskopische Vakuumtherapie, Anastomoseninsuffizienz, Risikoanastomose, Prophylaxe, Prävention, Intraluminal endoscopic vacuum therapy, Anastomosis insufficiency, At-risk anastomosis, Prophylaxis, Prevention

## Abstract

**Einleitung:**

Der früh postoperative Reflux (PR) kann die Anastomosenheilung nach Ivor-Lewis-Ösophagektomie (ILE) beeinträchtigen und stellt ein Risiko für Aspirationen dar. Die Anastomoseninsuffizienz ist die bedrohlichste chirurgische Komplikation.Wir stellen die protektive Methode der pre-emptiven aktiven Refluxdrainage (PARD) mit gleichzeitiger enteraler Ernährung vor. Wir berichten über unsere Erfahrungen mit dem neuen Sicherheitskonzept für die Ösophaguschirurgie an einem Patientenkollektiv von 43 Patienten.

**Materialien und Methoden:**

Zur PARD nutzen wir eine doppellumige offenporige Foliendrainage (dOFD). Zur Herstellung der dOFD wird der gastrale Schenkel einer Trelumina Sonde (Freka®Trelumina, Fresenius) über eine Länge von 25 cm mit einer doppellagigen offenporigen Drainagefolie (Suprasorb®CNP Drainagefolie, Lohmann & Rauscher) beschichtet. Die dOFD wird nach Fertigstellung der Anastomose intraoperativ endoskopisch im Schlauchmagen eingeführt. Es wird ein kontinuierlicher Unterdruck mit einer elektronischen Pumpe angelegt (−125 mm Hg). Der PR wird permanent komplett abgesaugt, der Magen und die Anastomosenregion dekomprimiert. Gleichzeitig wird über eine integrierte Intestinalsonde ernährt. Je nach den Ergebnissen der endoskopischen Kontrolle nach 5 Tagen wird PARD entweder fortgesetzt oder beendet.

**Ergebnisse:**

Im Beobachtungszeitraum (2017–2023) wurde PARD bei allen Patienten (*n* = 43) mit ILE eingesetzt. Die Heilungsrate unter PARD betrug 100 %, bei allen Anastomosen wurde eine Abheilung beobachtet. Es wurden keine zusätzlichen endoskopischen Eingriffe oder chirurgische Revisionen an den Anastomosen erforderlich. Die mediane Dauer der PARD betrug 8 Tage (Spanne 4–21). Bei 20 von 43 Patienten (47 %) sahen wir Probleme in der Anastomosenheilung, für die wir endoskopische Kriterien der Risikoanastomose definierten.

**Schlussfolgerung:**

Unsere Ergebnisse legen nahe, dass PARD einen starken protektiven Effekt auf die Anastomosenheilung hat und das Risiko der Anastomoseninsuffizienz reduzieren kann. Die integrierte Ernährungssonde der dOFD ermöglicht simultan zur Unterdruckausübung die frühpostoperative enterale Ernährung. PARD scheint die negativen Folgen einer gestörten Anastomosenheilung zu verhindern.

## Abstract (en)

**Introduction:**

Early postoperative reflux (PR) can compromise anastomotic healing after Ivor Lewis esophagectomy (ILE) and poses a risk for aspiration. Anastomotic insufficiency is the most threatening surgical complication. We present the protective method of pre-emptive active reflux drainage (PARD) with simultaneous enteral feeding. We report our experience with this new safety concept in esophageal surgery in a cohort of 43 patients.

**Materials and Methods:**

For PARD we use a double lumen open porous film drainage (dOFD). To create the dOFD, the gastric tube of a Trelumina probe (Freka®Trelumina, Fresenius) is coated with a double-layered open-pore drainage film (Suprasorb®CNP drainage film, Lohmann & Rauscher) over a length of 25 cm. The dOFD is endoscopically inserted into the tubular stomach intraoperatively after completion of the anastomosis. Continuous negative pressure is applied with an electronic pump (−125 mm Hg). The PR is continuously aspirated completely and the stomach and anastomotic region are decompressed. At the same time, nutrition is delivered via an integrated intestinal tube. Depending on the results of the endoscopic control after 5 days, PARD is either continued or terminated.

**Results:**

During the observation period (2017–2023), PARD was used in all patients (*n* = 43) with ILE. The healing rate under PARD was 100% and healing was observed in all anastomoses. No additional endoscopic procedures or surgical revisions of the anastomoses were required. The median duration of PARD was 8 days (range 4–21). We observed problems in the healing of the anastomosis in 20 of 43 patients (47%) for whom we defined endoscopic criteria for at-risk anastomosis.

**Conclusions:**

Our results suggest that PARD has a strong protective effect on anastomotic healing and may reduce the risk of anastomotic insufficiency. The integrated feeding tube of the dOFD allows early postoperative enteral feeding while simultaneously applying negative pressure. PARD appears to prevent the negative consequences of impaired anastomotic healing.

## Einführung

Die abdominothorakale Ösophagusresektion ist die chirurgische Therapieoption bei operablen Karzinomen des gastroösophagealen Übergangs (AEG Typ I–II). Stadienadaptiert wird präoperativ eine neoadjuvante onkologische Vorbehandlung durchgeführt. Das größte Risiko im postoperativen Verlauf ist das Auftreten einer Heilungsstörung der neu angelegten ösophagogastralen Anastomose. Die Anastomoseninsuffizienz ist die bedrohlichste chirurgische Komplikation nach abdominothorakaler Ösophagusresektion [1]. Durch die intrathorakale Lage der Anastomose sind die Patienten neben der lokalen Infektion insbesondere durch eine septische Mediastinitis mit hoher Morbidität und Mortalität bedroht.

Verschiedene Faktoren sind für die Entstehung einer Anastomoseninsuffizienz relevant, und eine Vielzahl von Strategien zur Verhinderung dieser bedrohlichen Komplikation lassen sich beschreiben [2]. Technisch-chirurgische Voraussetzungen sind beispielsweise eine möglichst spannungsfreie Anastomosierung und die Sicherungen einer guten Durchblutung. Es zeigt sich allerdings, dass auch bei optimalen Bedingungen, wie bei Durchführung der Operation in einem High-volume-Zentrum, durch die Einführung von minimal-invasiven Operationstechniken und robotergeführten oder Hybridoperationen [3] und durch intraoperative Überprüfung der Perfusion durch Fluoreszenz [4] etc., unverändert ein relevantes Risiko für das Auftreten einer Anastomoseninsuffizienz besteht [2]. Die Inzidenz ist hoch und erreicht auch in aktuellen Studien einzelner Zentren eine Rate von 25 % [5, 6].

Die Höhe der Komplikationsrate nach Ösophagektomie korreliert dabei nicht mit der Fallzahl der in einem Hause durchgeführten Operationen. Entscheidend für gute Behandlungsergebnisse scheint ein kompetentes multidisziplinäres perioperatives Komplikationsmanagement beim Auftreten postoperativer Probleme zu sein [7–10]. Eine besondere Bedeutung kommt hier der interventionellen Endoskopie zu [8].

**Figure 1:**
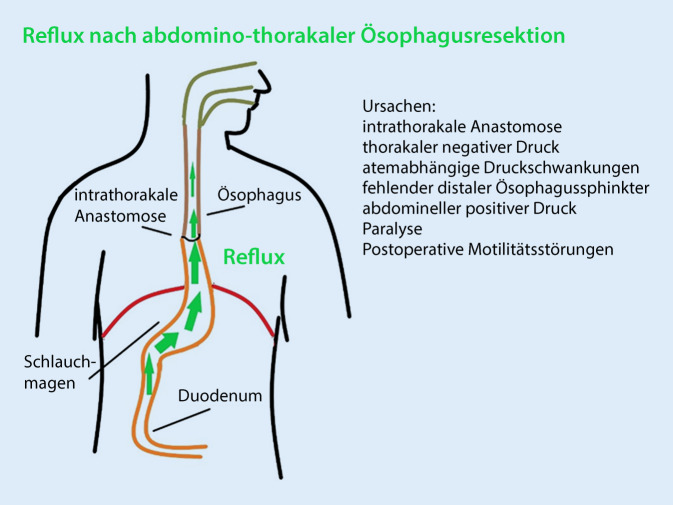

Bei der Mehrzahl der abdominothorakalen Ösophagektomien kommt es aufgrund der veränderten postoperativen Anatomie zu einem Reflux [11, 12]. Die Ursachen hierfür sind der fehlende Ösophagusverschluss durch die Resektion des distalen Ösophagussphinkters, die intrathorakale Lage der Anastomose, das Druckgefälle mit intrathorakalem Unterdruck und positivem Abdominaldruck sowie die postoperative Paralyse (Abb. 1; Tab. 1). Der postoperative Reflux (PR) kann die Anastomosenheilung nach Ivor-Lewis-Ösophagektomie (ILE) beeinträchtigen und stellt darüber hinaus ein relevantes Risiko für Aspirationen dar.

**Table 1:** 

Ursachen des frühpostoperativen Refluxes nach Ösophagektomie
Intrathorakale Anastomose
Thorakaler Unterdruck
Atemabhängig intrathorakale Unterdruckschwankungen
Fehlender distaler Ösophagussphinkter
Abdomineller positiver Druck
Paralyse
Postoperative Motilitätsstörungen

In den postoperativ routinemäßig durchgeführten endoskopischen Kontrolluntersuchungen lässt sich beobachten, dass trotz einer einliegenden Magenableitsonde das gallige Refluxsekret die Anastomosenregion überspült. Dieses führt dazu, dass in der frühpostoperativen Heilungsphase die Anastomose permanent den enzymatisch wirksamen Verdauungssekreten ausgesetzt ist, die ihre Verdauungsaktivität an der frischen Anastomose entfalten. Der lokale Heilungsprozess kann hierdurch gefährdet sein. Als endoskopisches Bild ist typischerweise eine grüngefärbte Anastomosenwunde sichtbar. Bei der endoskopischen Unterdrucktherapie (ENPT) von Anastomoseninsuffizienzen ließ sich beobachten, dass unter der aktiven Absaugung des Refluxes diese Veränderungen nicht mehr beobachtet werden und diese Drainage einen wesentlichen Beitrag zur Abheilung der Anastomose zu leisten scheint [13].

Auf der Grundlage der oben genannten klinischen Befunde und unserer zahlreichen Erfahrungen in der Anwendung der ENPT inauguierten wir die Methode der präemptiven aktiven Refluxdrainage (PARD) mit gleichzeitiger enteraler Ernährung. Die Methode stellt eine Weiterentwicklung der intraluminalen ENPT zur präemptiven Anastomosenprophylaxe dar. Seit 2017 wenden wir die PARD bei allen Patienten mit Ivor-Lewis-Ösophagektomie an. Unsere Erfahrungen bei der Anwendung bei 24 Patienten haben wir bereits dargestellt [14].

Mit dieser Arbeit berichten wir über die PARD in einem größeren Patientenkollektiv von 43 Patienten.

## Material und Methodik

Eingeschlossen in unsere retrospektive Beobachtungsstudie wurden alle Patienten, die sich im Beobachtungszeitraum von 11/2017 bis 4/2023 im Marienkrankenhaus einer Ösophagektomie nach Ivor-Lewis unterzogen haben. Seit 11/2017 ist die PARD integrativer Bestandteil unseres Behandlungskonzeptes.

**Figure 2:**
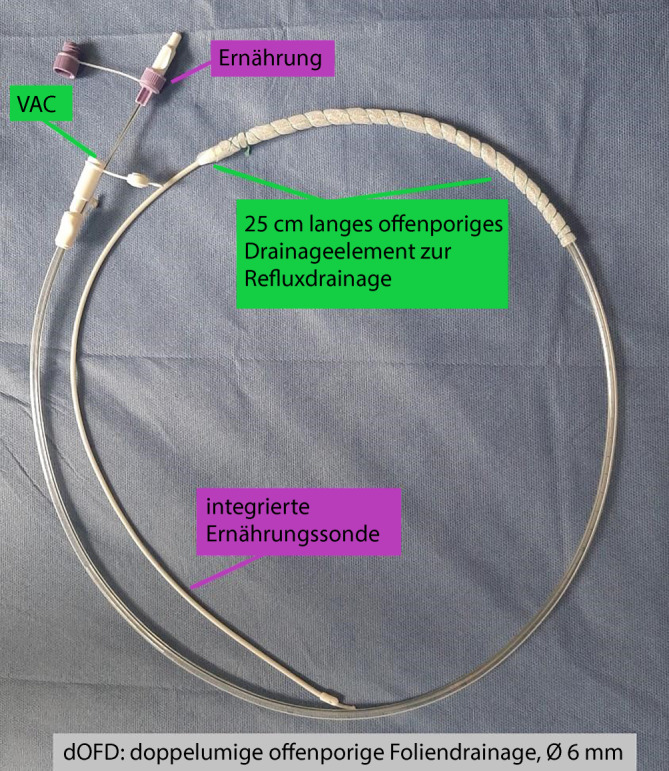

Zur PARD wird eine dünne doppellumige offenporigen Foliendrainage (dOFD) mit integrierter Ernährungssonde benutzt. Zur Herstellung wird der gastrale Abschnitt einer Trelumina-Sonde (Freka®Trelumina, Fresenius Kabi Deuschland GmbH, Bad Homburg, Deutschland) über eine Länge von 25 cm mit einer doppellagigen offenporigen Folie (Suprasorb®CNP Drainagefolie, Lohmann & Rauscher International GmbH & Co. KG, Rengsdorf, Deutschland) ummantelt. Um den Durchmesser der Trelumina-Sonde nicht wesentlich zu vergrößern, wird eine einlagige Folienumwicklung angestrebt. Je nach Breite des verwendeten Folienstreifens kann diese stellenweise auch mehrlagig ausfallen. Dieses beeinträchtigt nicht die Funktion, führt aber zu einem größeren Durchmesser des Sondenabschnittes. Der Belüftungskanal der Trelumina-Sonde wird mit einer Pressklemme verschlossen und hierdurch in seiner Funktion ausgeschaltet. Bei der PARD werden zwei Kanäle der Trelumina-Sonde benutzt (folienummantelter gastraler Ableitungskanal und der Ernährungskanal). Die dOFD hat einen Durchmesser von nur 6 mm. (Abb. 2 und 3).

**Figure 3:**
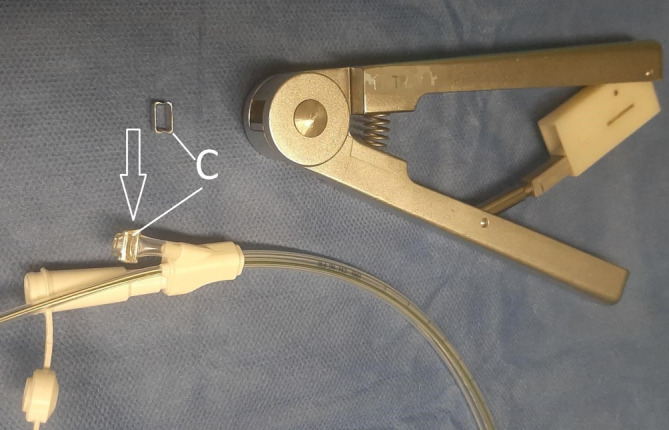

Intraoperativ, nach Fertigstellung der Anastomose, wird die dOFD transnasal, in gleicher Technik wie eine Magenableitsonde (NGT), eingeführt [13]. Die dOFD ersetzt sozusagen die Anlage einer NGT.

**Figure 4:**
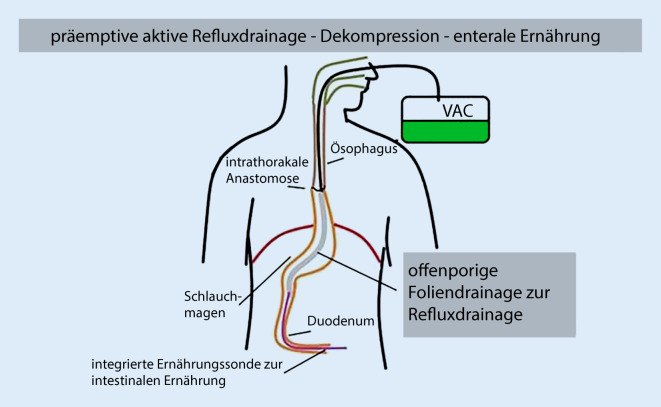

Unter endoskopischer Kontrolle wird das 25 cm lange folienbeschichtete Drainageelement (FDE) im Schlauchmagen positioniert. Das proximale Ende des FDE kommt distal der Anastomose zum Liegen. Das FDE erstreckt sich über die gesamte Länge des Schlauchmagens. Es reicht mit dem distalen Ende in der Regel bis zum präpylorischen Antrum oder sogar transpylorisch mit der Spitze ins proximale Duodenum. Die integrierte enterale Ernährungssonde (iT) wird tief über das Duodenum ins Jejunum vorgeschoben. Mittels einer elektronischen Vakuumpumpe (ACTIV.A.C; KCI, San Antonio, TX, USA) wird ein kontinuierlicher Unterdruck von −125 mm Hg an das FDE angelegt. Hierdurch wird der Magen permanent abgesaugt und dekomprimiert. Gleichzeitig erfolgt über die iT die enterale Ernährung mit Sondenkost. Wir beginnen am 1. postoperativen Tag mit Wasser und steigern hiernach am 2. bis 3. Tag auf Sondenkost. An die Anastomose wird kein Unterdruck angelegt. (Abb. 4).

**Figure 5:**
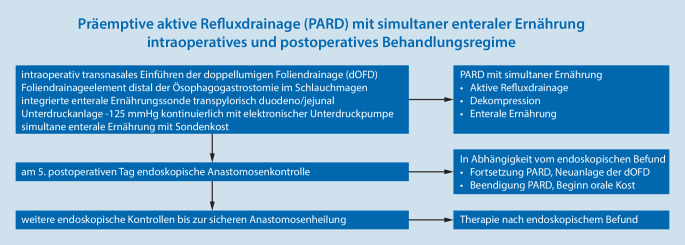

Unser Regime (Abb. 5) sieht vor, dass frühpostoperativ nach 5 Tagen routinemäßig eine endoskopische Kontrolle der Anastomose vorgenommen wird. Je nach Befund wird die PARD fortgesetzt oder beendet. Bei reizlosen Wundverhältnissen wird die PARD beendet und es folgt der orale Kostaufbau. Beim Vorliegen einer Risikoanastomose („at risk anastomosis“, ARA) wird die PARD fortgeführt. Die dOFD wird im Abstand von 3 bis 4 Tagen gewechselt bis nach endoskopischem Befund eine sichere Anastomosensituation vorliegt. In jedem Fall führen wir auch nach Abschluss der PARD weitere endoskopische Verlaufskontrollen durch, um die endgültige Abheilung zu sichern. Alle Patienten erhalten postoperativ eine medikamentöse Säureblockade i.v. in doppelter Dosierung.

Als ARA definieren wir Anastomosen, bei denen in der ersten postoperativen Kontrolle die folgenden endoskopischen Zeichen einer gestörten Wundheilung vorliegen: breite Ulzeration, Zeichen der Ischämie, Nekrosen, freiliegende Klammern oder sichtbares Nahtmaterial (Tab. 2).

**Table 2:** 

Endoskopische Kriterien von Risikoanastomosen
Freiliegende Kammern
Sichtbares Nahtmaterial
Breite Ulzerationen
Nekrosen
Zeichen der Ischämie

## Ergebnisse

Im Beobachtungszeitraum (11/2017–4/2023) wurde PARD bei allen Patienten (*n* = 43) (Altersspektrum von 53–80 Jahren, Median = 66 Jahre; 35 m, 8/f) mit ILE eingesetzt. 41 wurden in offener Technik operiert, 2 in Hybridtechnik. 36 von 43 Patienten wurden neoadjuvant therapiert.

Die endoskopische Platzierung der dOFD erfolgte intraoperativ nach Fertigstellung der Ösophagogastrostomie. Gleichzeitig zur Unterdruckausübung wurde über die integrierte Ernährungssonde bereits frühpostoperativ mit der enteralen Ernährung begonnen.

Die erste endoskopische Anastomosenkontrolle fand im Median nach 4 Tagen (2–7) statt. Bei allen Kontrollendoskopien fand sich ein entleerter und dekomprimierter Magenschlauch. Die Anastomosen waren frei von den Verdauungssekreten des Refluxes. Als endoskopisch sichtbare Zeichen stellten sich die Anastomosen weißlich hell gezeichnet und nicht grünlich inbibiert dar. Bei keinem Patienten wurde unter der PARD eine Aspirationspeumonie beobachtet.

Bei 20 von 43 Patienten (47 %) sahen wir Störungen bei der Anastomosenheilung, die wir als „Risikoanastomose“ („at risk anastomosis“, ARA) definieren (Tab. 2). Bei diesen Patienten wurde die PARD fortgeführt, bis nach endoskopischem Befund eine sichere Anastomosenheilung vorlag. Es wurden keine Reoperationen an der Anastomose erforderlich. Es wurden auch keine anderen endoskopischen Interventionen wie z. B. eine ENPT mit Polyurethanschaumdrainagen und direkter Unterdruckausübung an der Anastomose oder eine Therapie mittels selbstexpandierenden Stents oder Vakuumstents notwendig. 6 der 20 Patienten mit einer Risikoanastomose (30 %) entwickelten eine postoperative Anastomosenstenose (14 % des Gesamtkollektivs). Mittels Ballondilatation war diese leicht zu behandeln. Ein Patient verstarb 18 Tage nach der Operation an einer Pneumonie.

Bei allen Anastomosen (100 %) wurde unter PARD eine Abheilung beobachtet. Die mediane Dauer der PARD betrug 8 Tage (4–21; Tab. 3).

**Table 3:** 

Patient	Geschlecht	Alter (a)	Neoadjuvante Therapie	Tumor	Anastomosenhöhe in cm (von der Zahnreihe)	Insertion der OFD (*n*)	Erste Endoskopie (postop. Tag)	Dauer der EVT (d)	Anastomosenheilung	Risikoanastomose (ARA)	Zusätzliche Therapie an der Anastomose?	Stenose	Dilatation
1	m	75	FLOT	ypT3 yPN1	30	1	7	7	+	–	n	n	n
2	m	53	FLOT	ypT3 ypN1	29	2	4	11	+	ARA	n	n	n
3	m	75	FLOT	ypT2 pN0	25	2	6	10	+	ARA	n	n	n
4	f	53	FLOT	ypT0 ypN0	15	2	4	8	+	–	n	n	n
5	m	63	–	pT1 pN0	25	1	4	4	+	–	n	n	n
6	f	72	–	pT1 pN0	19	1	4	8	+	–	n	n	n
7	m	62	FLOT	ypT3 ypN1	25	7	2	21	+	ARA	n	+	+
8	m	73	FLOT	ypT2 pN0	25	6	2	19	+	ARA	n	n	n
9	f	79	CROSS	ypT0 pN0	23	2	4	7	+	ARA	n	n	n
10	f	61	FLOT	ypT3 pN1	23	2	3	10	+	–	n	n	n
11	m	67	FLOT	ypT3 yPN2	25	2	3	8	+	ARA	n	n	n
12	m	64	FLOT	ypT0 pN0	25	4	4	17	+	–	n	n	n
13	m	78	–	pT2 pN1	25	2	3	8	+	–	n	n	n
14	m	64	–	pT3 pN2	25	2	4	7	+	–	n	n	n
15	m	77	FLOT	ypT3 pN1	24	4	4	10	+	ARA	n	n	n
16	f	66	–	pT1b pN0	22	2	6	10	+	ARA	n	+	+
17	m	59	FLOT	ypT3 pN1	26	2	2	7	+	–	n	n	n
18	m	61	FLOT	ypT0 pN0	27	1	4	4	+	–	n	n	n
19	f	75	FLOT	ypT3 pN2	25	4	4	15	+	–	n	n	n
20	m	68	FLOT	ypT3 pN0	25	5	4	15	+	ARA	n	n	n
21	m	68	FLOT	ypT2 pN1	25	2	3	8	+	–	n	n	n
22	m	77	FLOT	ypT3 pN0	25	5	1	13	+	ARA	n	+	+
23	m	72	FLOT	ypT0 pN0	24	2	4	7	+	–	n	n	n
24	m	64	–	pT3 pN0	24	2	4	7	+	–	n	n	n
25	f	67	–	pT1b pN0	24	2	2	5	+	ARA	n	n	n
26	m	65	FLOT	ypT0 pN0	25	4	1	14	+	ARA	n	+	+
27	m	60	FLOT	ypT3 pN0	26	1	5	5	+	ARA	n	+	+
28	m	58	FLOT	ypT3 pN3	25	1	4	4	+	–	n	n	n
29	m	80	FLOT	ypT3 pN1	24	2	1	7	+	–	n	n	n
30	m	73	FLOT	yPT0 pN0	25	1	4	4	+	–	n	n	n
31	m	65	FLOT	ypT3 pN2	25	3	4	12	+	ARA	n	n	n
32	m	83	–	pT1 pN0	28	3	2	10	+	–	n	n	n
33	m	51	FLOT	ypT0 pN0	25	3	5	12	+	ARA	n	+	+
34	m	49	FLOT	ypT2 pN0	25	1	4	4	+	–	n	n	n
35	m	60	–	pT1b pN0	25	1	6	6	+	–	n	n	n
36	m	64	FLOT	ypT3 pN3	25	2	2	5	+	–	n	n	n
37	m	63	FLOT	ypT3 ypN1	25	2	5	8	+	ARA	n	n	n
38	m	70	CROSS	ypT0 ypN1	25	4	5	18	+	ARA	n	n	n
39	m	61	CROSS	ypT3 ypN1	29	2	2	6	+	–	n	n	n
40	f	66	–	pT1b pN0	25	1	7	12	+	ARA	n	n	n
41	m	74	FLOT	ypT1b ypN1	25	1	3	7	+	–	n	n	n
42	m	59	FLOT	ypT3 ypN1	25	3	3	12	+	ARA	n	n	n
43	m	79	FLOT	ypT1a ypN0	20	3	5	11	+	ARA	n	n	n
	*35m; 8f*	*m* *=* *66 a (53–80)*			*m* *=* *25* *cm*	*m* *=* *2*	*m* *=* *4*	*m* *=* *8*	*Heilung 43/43* *=* *100* *%*	*n* *=* *20*		*n* *=* *6*	*n* *=* *6*

^a^Die Heilungsrate der Anastomosen liegt bei 100 %. Es wurden keine Reoperationen und keine anderen endoskopischen Behandlungen in der Heilungsphase notwendig. Ein Patient verstarb 18 Tage nach der Operation an einer Pneumonie

## Diskussion

Die abdominothorakale Ösophagusresektion gehört zu den morbiditätsträchtigen Eingriffen in der onkologischen Chirurgie. Auch bei sorgfältigster Beachtung der bekannten Strategien zur Vermeidung von Komplikationen [2] besteht ein relevantes Risiko für das Auftreten einer manifesten Anastomoseninsuffizienz. Diese Heilungsstörung stellt die größte Gefährdung für die Patienten dar.

In der Komplikationsdiagnostik sind in den letzten Jahren erhebliche Fortschritte erzielt worden. Die endoskopische Inspektion der Nahtstelle ist das wichtigste Verfahren zum Ausschluss oder Bestätigung einer Abheilungsstörung. In direkter Sicht ermöglicht sie die Bestimmung der Ursache, exakte Beschreibung des Größenausmaßes und der Durchblutungsverhältnisse. Im Idealfall kann ohne Verzögerung bereits unmittelbar nach der Diagnose in derselben Untersuchung die notwendige endoskopisch-therapeutische Maßnahme zur Unterbrechung der Kontamination vorgenommen werden. Ergänzt wird die Endoskopie von der Computertomographie, die zusätzliche Informationen zu den extraluminalen thorakalen Gegebenheiten liefern kann [15].

In der Therapie der Anastomosenheilungsstörung ist ein deutlicher Wandel in der chirurgischen Behandlung zu konstatieren. Operative Ansätze wie die Diskontiniutätsresektion mit Ausleitung des proximalen Ösophagus als Speichelfistelung und Blindverschluss des ableitenden Magens werden nur noch extrem selten durchgeführt. Ganz im Vordergrund der Behandlung stehen notfallmäßige endoskopisch-interventionelle Verfahren, die initial zur Kontrolle bzw. Verhinderung des Auftretens einer Mediastinitis eingesetzt werden.

Ein besonders hoher Stellenwert kommt hierbei der endoskopischen Unterdrucktherapie zu, die heute in unserer Region als Therapie der ersten Wahl angesehen werden kann. Der Behandlungserfolg liegt bei ca. 85 %. In den meisten Fällen gelingt es, die Anastomoseninsuffizienzen endoskopisch zu therapieren und Revisionsoperationen zu vermeiden. Die Kenntnis und Beherrschung dieser endoskopischen Intervention hat in den letzten Jahren einen festen Stellenwert im Komplikationsmanagement nach Ösophaguseingriffen erlangt und stellt eine wichtige Voraussetzung dar, um den Patienten das risikoreiche Operationsverfahren mit einer großen Sicherheit anbieten zu können [15].

Es ist bemerkenswert, dass die modernen endoskopischen Techniken durch chirurgische Kliniken vorangetrieben werden, die sowohl über die operative Expertise verfügen als auch die endoskopischen Verfahren zur Komplikationsbehandlung beherrschen [16]. Die Entwicklung der ENPT am oberen Gastrointestinaltrakt wurde seit 2006 maßgeblich von der chirurgisch-endoskopischen Arbeitsgruppe des Marienkrankenhauses initiiert. In mittlerweile mehr als 50 Publikationen konnte über die variantenreiche Therapieform am oberen Gastrointestinaltrakt in zahlreichen Erstbeschreibungen, retrospektiven Fallserien, Videopublikationen sowie Weiterentwicklungen von technischen Materialien berichtet werden (www. Endoscopicvacuumtherapy.com). Die hier vorgestellte Methode der PARD hat sich aus den Erfahrungen der zahlreichen klinischen Anwendungen der ENPT entwickelt.

Anfänglich wurde die ENPT zur Behandlung der eingetretenen schweren Komplikation angewendet. 2014 berichteten wir an einer Kohorte von bereits 35 Patienten, wie sich die ENPT zur Komplikationsbehandlung am Ösophagus nutzen lässt. Die Insuffizienzrate in unserer Kohorte betrug damals 17 % [17]. Mit der Weiterentwicklung der Drainagen- und Platzierungstechnik und der Erkenntnis, dass sich refluierende Duodenalsekrete optimal mittels ENPT ableiten lassen sowie der Inauguration der intraluminalen Variante der endoskopischen Unterdrucktherapie [13] entwickelten wir schrittweise das Konzept der PARD. Die Verwendung der dünnen offenporigen Drainagefolie (Suprasorb CNP-Drainagefolie, Lohmann &Rauscher) ermöglichte die Entwicklung einer innovativen dünnen Unterdruckdrainage (OFD) für die ENPT, die wie eine Magenableitsonde („naso-gastric tube“, NGT) gehandhabt werden kann [18].

Vereinfacht könnte man sagen, dass die OFD eine NGT ist, an die ein Unterdruck angelegt werden kann. Der Unterschied zu einer NGT besteht in der vollständigen Magenentleerung, die bei einer passiven NGT nicht gegeben ist. Bei passiven Drainagen wie NGTs erfolgt die Entleerung entlang der Schwer- und Kapillarkraft und bei Überdruck. In zahlreichen endoskopischen Anastomosenkontrollen haben wir beobachtet, dass die Anastomosenregion trotz korrekt liegender passiver NGT vom Reflux überspült wird. Dieses Phänomen der unvollständigen Magenentleerung beim Vorhandensein einer NGT werden Endoskopiker, die diese Patienten untersuchen, leicht bestätigen können.

Unser Therapiealgorithmus sieht eine frühe Kontrollendoskopie nach 5 Tagen vor. Im Einzelfall wurde hiervon abgewichen. Ein Grund für eine frühere Untersuchung ist beispielsweise die Entwicklung eines auffälligen Infektlabors. Längere Sondeneinlagen haben wir im Einzelfall bei fehlender Endoskopiekapazität oder Wechselintervall am Wochenende toleriert. Wenn der Reflux abgesaugt wird, kann man von einer funktionsfähigen dOFD ausgehen. Eine nicht ausreichende Saugleistung haben wir initial nie beobachtet. Aber immer dann, wenn kein Reflux gefördert wird, muss auch die Funktionslosigkeit der Sonde z. B. durch eine Verstopfung der Folie in Betracht gezogen werden. Dieses kann nur durch einen Wechsel der Sonde kontrolliert und behoben werden. Andere Fehler können auch ein Unterbleiben des Wechsels voller Kanister oder eine Funktionsstörung der Pumpe sein.

Das Wissen um die Risiken für eine Anastomosenheilung und Kenntnisse über die geänderte Physiologie des neuen Verdauungstraktes haben zu einer Weiterentwicklung der endoskopischen Unterdrucktherapie hin zur neuen minimal-invasiven Methode der präemptiven Anwendung geführt. Das dahinterstehende Behandlungsprinzip ist einfach. Die aggressiven Sekrete des postoperativen Refluxes (PR) werden in der vulnerablen frühen Phase der Wundheilung von der Ösophagogastrostomie ferngehalten. PR enthält gastrale, pankreatische, biliäre, duodenale und orale enzymatische Sekrete, deren physiologische Aufgabe der Verdauung dienen. PR induziert eine Ösophagitis und erhöht das Risiko der pulmonalen Aspiration. Die aktive Entleerung führt darüber hinaus zu einer Dekompression des Magenschlauches und der Anastomosenregion. Simultan ermöglicht eine Ausrüstung der dOFD mit einer integrierten Ernährungssonde die sehr frühzeitige enterale Ernährung. Die operative Anlage einer Jejunostomie, die ebenfalls ein eigenes Risikoprofil hat, erübrigt sich [19].

Seit 2017 wenden wir diese Sicherheitstechnik bei allen Patienten mit abdominothorakaler Ösophagusresektion an. Auch nach Chefarztwechsel im April 2023 wird die Methode unter neuer Klinikleitung auch im Rahmen der robotischen Ösophaguschirurgie fortgeführt. Seit der Einführung der PARD liegt die Heilungsrate der Anastomosen nach Ösophagektomie im Marienkrankenhaus bei 100 %. 43 Patienten haben wir seit der Einführung der Methode präemptiv behandelt. Unter der Annahme einer Insuffizienzrate von 17 % hätten wir ohne PARD bei ca. 7 der 43 Patienten eine Anastomoseninsuffizienz erwartet. Bei fast der Hälfte unserer Patienten (47 %) sahen wir eine Störung der Wundheilung, für die wir Kriterien einer Risikoanastomose definierten. Diese Zahl scheint auf den ersten Blick hoch zu sein, korreliert unseres Erachtens jedoch gut mit der Inzidenz einer zu erwartenden Anastomoseninsuffizienzrate, wenn keine PARD vorgenommen werden würde. Wir gehen davon aus, dass ein wesentlicher Teil dieser Risikopatienten eine manifeste Anastomoseninsuffizienz entwickelt hätte. Besonders die frühpostoperative Sichtbarkeit von Klammern und Nahtmaterial werten wir als ein wichtiges Alarmzeichen. Müller et al. haben eine ähnliche Risikobeschreibung der postoperativen Anastomosen vorgenommen, sie versuchen das Ausmaß der Einschränkung noch feiner zu beschreiben [23]. Unsere Risikodefinition ist pragmatischer und einfach (Tab. 2). Immer wenn eines der Kriterien anzutreffen ist, erfolgt die Fortführung der PARD [14]. Aus Gründen der erhöhten Sicherheit nehmen wir in Kauf, dass ein Teil der Patienten durch eine Verlängerung der PARD und das Belassen der nasalen dOFD eine mögliche Übertherapie erfährt.

Die Rate der Anastomosenstenose erklärt sich aus den beschriebenen Kriterien der Wundheilungsstörung bei Risikoanastomosen. Sie sind nicht Folge einer prolongierten PARD. Nach unseren zahlreichen Erfahrungen in der endoskopischen Anastomosenkontrolle, auch an anderen Lokalisationen wie beispielsweise im Rektum, haben insbesondere diejenigen Risikoanastomosen ein erhöhtes Stenoserisiko, bei denen eine Ischämie zu beobachten war. Patienten mit einer ARA binden wir in einem längeren postoperativen Überwachungsintervall endoskopisch an. Alle Stenosen waren einfach zu behandeln.

Ein weiterer wichtiger Sicherheitseffekt der Methode ist die Vermeidung von frühpostoperativen Aspirationen durch Reflux, die bei fehlendem Sekret nicht stattfinden.

Prinzipiell könnte die PARD anstelle folienbeschichter Drainagen auch mit offenporigen Polyurethanschaum beschichten Unterdruckdrainagen (OPD) vorgenommen werden. Allerdings sind hierfür spezielle endoskopische Einführtechniken notwendig. Ein Nachteil der derzeit kommerziell verfügbaren OPD ist der voluminöse Durchmesser des Drainageelements und die fehlende Ernährungssonde. Die integrierte Intestinalsonde der dOFD ermöglicht die sehr frühpostoperativ beginnende Ernährung simultan zur Unterdruckbehandlung ohne Jejunostomie. Einen großen Vorteil in der Handhabung der dOFD sehen wir in dem minimale Drainagendurchmesser, der die direkte transnasale Einführung möglich macht. Der Durchmesser der Trelumina-Sonde nimmt durch die Folienummantelung minimal zu, dieses lässt sich durch die individuelle händische Anfertigung nicht vermeiden. Gelegentlich haben wir beim Einführen und Wechsel der dOFD eine Epistaxis beobachtet. Eine Behandlungsbedürftigkeit durch HNO bestand nie. Es ist davon auszugehen, dass sich bei einer industriellen Fertigungsmöglichkeit Foliensonden mit einem noch geringeren Durchmesser herstellen lassen. Bei einlumigen Foliensonden erreichen wir Sondendurchmesser von 4 mm.

PARD beschränkt sich auf die intragastrale Unterdruckausübung mit dem Ziel der Entfernung des gastralen Refluxes. Die Anastomose selbst wird nicht mit dem Unterdruck behandelt. Dies wäre leicht durch eine proximalere Positionierung des FDE oder Verlängerung des FDE möglich. Folienbasierte Drainagen haften unter Sog weniger fest dem anliegenden Gewebe an als OPD.

Die präemptive postoperative intraluminale Anwendung der ENPT mit OPD bei Risikoanastomosen zeigten Neumann et al. in einer kleinen Fallserie. Alle Anastomosen kamen zur Abheilung [21]. Eine tierexperimentelle Studie unterstützt diese Beobachtung. Trotz intraoperativ belassenem Anastomosendefekt heilten die Anastomosen unter intraoperativ eingeleiteter ENPT mit einer intraluminalen Platzierung der OPD über der Anastomosenregion [22, 23]. Einzelne Gruppen sind hiernach dazu übergegangen, den intraoperativ beginnenden präemptiven Einsatz von OPD mit Installierung der Unterdruckausübung über der Anastomose klinisch vorzunehmen. Es wurden vergleichbar gute Ergebnisse berichtet [20, 24]. Im Einsatz bei Hochrisikoanastomosen bei Revisionsoperationen konnten Anastomoseninsuffizienzen nicht verhindert werden, dennoch wird der Einsatz als hilfreich angesehen [25]. Auch für die Anwendung des polyurethanbeschichteten selbstexpandierenden Stents in präemptiver Indikation liegen erste klinische Ergebnisse vor [26]. Eine erste systematische Metaanalyse über den präemptiven Einsatz der ENPT auch für andere Indikationen nach Operationen am Gastrointestinaltrakt liegt bereits vor [27]. Auch für Rektumanastomosen soll der Nutzen der präemptive ENPT überprüft werden [28].

**Figure 6:**
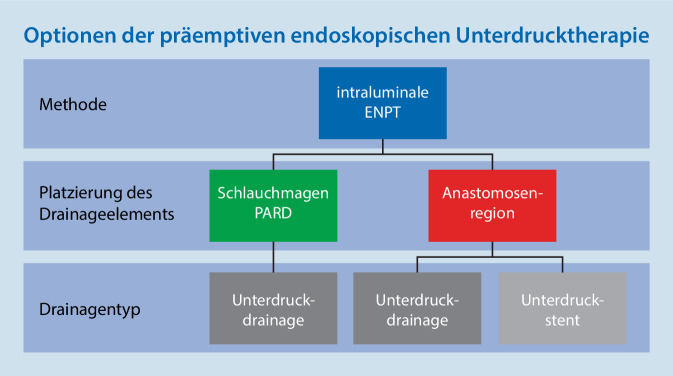

Eine 2015 initiierte Studie zur Frage, ob eine intraluminale Unterdrucktherapie mit Schaumdrainagen die Rate an Anastomoseninsuffizienzen senken kann, konnte nicht umgesetzt werden. Eine weitere Studie mit gleicher Fragestellung ist initiiert [29]. Es wäre von Interesse, die präemptive Nutzung der ENPT in verschiedenen denkbaren Ausführungsformen prospektiv zu vergleichen. Die folgenden Methoden lassen sich definieren, sie unterscheiden sich in dem verwendeten Drainagematerial, dem Platzierungsort und Ausführungen der Drainagen. Infrage kommen die alleinige intragastrale Refluxdrainage (PARD), die intragastrale Refluxdrainage in Kombination mit direkter intraösophagealer Unterdruckausübung auf der Anastomosenregion und die Unterdruckausübung auf der Anastomosenregion mit einem selbstexpandierenden Unterdruckstent (Abb. 6). Bei allen Varianten ist die Ausrüstung der Drainagen mit der offenporigen Drainagefolie oder Polyurethanschaum sowie mit oder ohne Ernährungssonde möglich.

Unter den verschiedenen Möglichkeiten halten wir die PARD für die technisch einfachste Methode mit nachgewiesenem hohem Nutzen. Sie ist fester Bestandteil unseres Behandlungsregimes.

**Figure 7:**
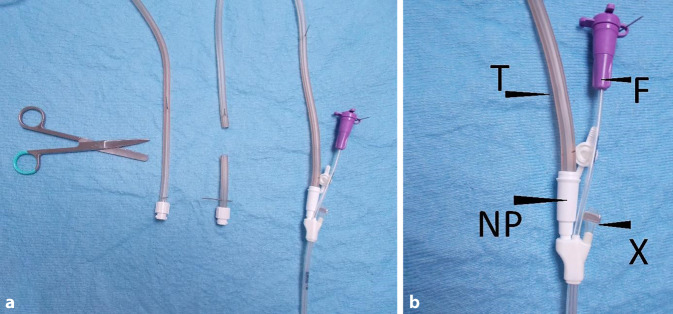

Zur Einführung der neuen Methode sind Vorbereitungen sowohl für die eigentliche Durchführung als auch für die postoperative Versorgung zu treffen, die wir kurz skizzieren. Ärzt*innen und Pflegepersonal im Operationssaal, auf Intensiv- und Normalstation sowie Anästhesie und Endoskopie müssen informiert sein. In unserem Ablauf hat sich bewährt, die Sondenplatzierung nach Abschluss der eigentlichen Operation in Rücklage am noch intubierten Patienten vorzunehmen. Dieses kann im Operationssaal oder auch vor der Extubation auf der Intensivstation erfolgen. Die Anästhesie muss wissen, dass die Narkose aufgrund der Endoskopie noch für 15–30 min verlängert werden muss. Eine Unterdruckpumpe mit Kanister und die dOFD müssen bereitstehen. Die dOFD fertigen wir bereits vor dem Eingriff am Tag zuvor an. Nach Einsetzen des Beißschutzes wird mit einem diagnostischen Gastroskop in den Ösophagus eingespiegelt. Wir nutzen immer CO_2_ als Untersuchungsgas. Die zügige Untersuchung beschränkt sich auf eine vorsichtige grobe Inspektion. Es wird keine Schleimhautfeindiagnostik mit Magenüberblähung und Distension vorgenommen! Alle groben Bewegungen sind zu vermeiden. Eine Inversion im Schlauchmagen wird nicht vorgenommen. Die Anastomose wird inspiziert, die Durchblutungssituation eingeschätzt. Häufiger lassen sich Veränderung wie ein Ödem oder auch ein Hämatom darstellen. Im Schlauchmagen findet sich meist etwas blutiges Sekret, dieses wird gespült und abgesaugt. Über den Pylorus wird in das gestreckte Duodenum passiert. Das Endoskop wird entfernt. Der Mandrin der Ernährungssonde und die Sonde werden geölt. Anschließend wird die dOFD über ein Nasenloch eingeführt und unter endoskopischer Sicht in den zervikalen Ösophagus eingeschoben. Die Passage der Spitze der dOFD über die Anastomose sollte unter endoskopischer Kontrolle erfolgen. Das weitere Vorschieben der Sonde erfolgt in üblicher endoskopischer Technik mit Assistenz zusammen mit dem Endoskop, hierzu Fixierung an der Nase beim Rückzug des Endoskops. Die Ernährungssonde kann mit einer Greifzange gegriffen und durch den Pylorus vorgeschoben werden. Das folienbeschichtete Drainageelement wird im Schlauchmagen so tief platziert, dass es von der Anastomose bis zum Pylorus reicht. Ein Abdecken der Anastomose ist nicht intendiert, wäre aber leicht möglich. Unter digitaler Fixierung der Sonde an der Nase wird das Gastroskop entfernt, eine Schlaufenbildung im Pharynx ausgeschlossen. Wir fixieren die Sonde mit einer Naht an der Nasolabialfalte, um eine versehentliche Dislokation zu verhindert. Der gastrale folienarmierte Drainageschenkel wird mit dem Schlauch der Unterdruckpumpe verbunden und der Sog von −125 mm Hg angelegt. Der Mandrin der Ernährungssonde wird entfernt, die Durchgängigkeit geprüft. Die Beschickung mit Wasser kann unmittelbar postoperativ erfolgen. Aus dem klinischen Alltag wissen wir, dass die Herstellung der Verbindung dOFD zum Drainageschlauch immer wieder eine Schwierigkeit darstellt. Für die postoperative Betreuung muss diese einfache Prozedur dem Personal bekannt sein. Das Verbindungsstück am Schlauch zum Kanister der Pumpe wird abgeschnitten und anschließend in den Ansatztubus der dOFD gesteckt. Diese Verbindung ist ausreichend, sie kann zusätzlich noch mit einem Pflaster gesichert werden (Abb. 7). Wenn der Kanister voll ist, wird der Kanister gewechselt. Nach ca. 5 Tagen erfolgt eine Anastomosenkontrolle. Wir tackten die Untersuchungsintervalle so, dass sie in den Wochenablauf der Endoskopie integriert sind.

Ein Nachteil der PARD-Methode, sowie auch aller anderen präemptiven Methoden, könnte darin gesehen werden, dass ein Teil der Patienten möglicherweise eine Übertherapie erfährt und die nasale Sonde einige Tage zu lange behält. Bei PARD sehen wir allerdings auch bei diesen Patienten zwei wesentliche Vorteile: zum einen verheilen die Anastomosen in der ersten vulnerablen postoperativen Phase entzündungsarm und zum anderen reduzieren wir durch die Refluxabsaugung das Risiko der Aspiration in der postoperativen Frühphase.

Die filmbasierten Drainagen setzen wir im klinischen Alltag in einem wesentlich breiteren Spektrum ein. Therapeutisch erweitern sie das Spektrum der ENPT bei zahlreichen Indikationen erheblich und ersetzen oder ergänzen schaumbasierte Drainagen (z. B. bei kleinen Defektöffnungen, Fisteln, im Duodenum, Urologie) [30–32]. Für den präemptiven Einsatz kommen alle Operationen mit Anastomosen im oberen GI-Trakt infrage.

## Schlussfolgerung

Die intrathorakale Anastomose ist die Achillesferse in der frühen postoperativen Phase nach ILE. Der Anastomosenabheilung ist die größte Aufmerksamkeit zu widmen. Mit der endoskopischen Unterdrucktherapie steht ein Instrumentarium zur Verfügung, das eine aktive lokale Unterstützung der Wundheilung an der Anastomose ermöglicht.

Seit Einführung der präemptiven aktiven Refluxdrainage bei Ivor-Lewis-Ösophagektomie mit einer dOFD mit integrierter Ernährungssonde kam es in allen 43 Fällen zu einer suffizienten Abheilung der intrathorakalen Anastomosen. Es wurden keine Reoperationen an der Anastomose oder andere endoskopische Eingriffe notwendig. Die technisch einfache Methode ermöglicht eine enterale Ernährung bei gleichzeitiger permanenter Magenentleerung und -dekompression. Unsere Ergebnisse legen nahe, dass PARD einen starken protektiven Effekt auf die Anastomosenheilung hat und das Risiko der Anastomoseninsuffizienz reduzieren kann. Die integrierte Ernährungssonde der dOFD ermöglicht simultan zur Unterdruckausübung die frühpostoperative enterale Ernährung.

## Abkürzungen

AEG: Adenokarzinom des ösophagogastralen Übergangs
ARA: Risikoanastomose
dOFD: doppellumige offenporige Foliendrainage
ENPT: endoskopische Unterdrucktherapie
FDE: folienbeschichtetes Drainageelement
ILE: Ivor-Lewis-Ösophagektomie
iT: integrierte enterale Ernährungssonde
NGT: Magenableitsonde
OFD: offenporige Foliendrainage
OPD: offenporige Polyurethanschaumdrainage
PARD: präemptive aktive Refluxdrainage
PR: postoperativer Reflux
VAC: Unterdruck
